# Threat-Related Information Suggests Competence: A Possible Factor in the Spread of Rumors

**DOI:** 10.1371/journal.pone.0128421

**Published:** 2015-06-10

**Authors:** Pascal Boyer, Nora Parren

**Affiliations:** 1 Department of Psychology, Washington University in St. Louis, St. Louis, Missouri, United States of America; 2 Department of Anthropology, Washington University in St. Louis, St. Louis, Missouri, United States of America; 3 University of Lyon, Lyon, France; Center for BrainHealth, University of Texas at Dallas, UNITED STATES

## Abstract

Information about potential danger is a central component of many rumors, urban legends, ritual prescriptions, religious prohibitions and witchcraft crazes. We investigate a potential factor in the cultural success of such material, namely that a source of threat-related information may be intuitively judged as more competent than a source that does not convey such information. In five studies, we asked participants to judge which of two sources of information, only one of which conveyed threat-related information, was more knowledgeable. Results suggest that mention of potential danger makes a source appear more competent than others, that the effect is not due to a general negativity bias, and that it concerns competence rather than a more generally positive evaluation of the source.

## Introduction

Information about potential danger is a central element in many rumors [1,2], and urban legends [3–5], but also of ritual prescriptions [6], religious prohibitions [7] or witchcraft crazes [8,9]. Some of this cultural information has important social consequences, as for instance in witchcraft accusations but also in rumors about the alleged dangers of vaccination or medication [10,11]. This cultural spread begs the question of the individual processes whereby people attend to such information, but also find it compelling and relevant. Here we investigate one possible component of this prevalence of threat-related information, namely that all else being equal, a source of threat-related information may be intuitively judged as more *competent* than a source that does not convey such information, thereby increasing the motivation to transmit the negative message information.

There are of course a number of ways that a source of information or a potential partner could signal competence. So why focus on threat-information? Our conjecture is grounded in very general features of the psychology of threat-detection and precaution. While fear-systems guide responses to imminent danger, we also have dedicated systems to orient behaviors towards potential threats [12]. The activation of such systems may explain why humans are generally more attentive, all else being equal, to potential danger than potential benefits, a tendency variously described as a “negativity bias” [13–15] following which “bad is stronger than good” [16]. Several recent studies have demonstrated a specific consequence of this very general bias, namely that people seem to judge negatively framed pieces of information, (e.g. “5% of heart-attacks are lethal”) as more plausible than positively framed, identical information (e.g. “95% of heart-attack are not lethal”) [17–19].

In the studies presented here, we asked participants to judge which of two sources of information about various topics seemed more authoritative about various familiar situations. The only difference between the pieces of information conveyed by these two sources lay in the presence or absence of threat-related information, operationalized in these studies as the mention of potential danger.

### Ethics Statement

All studies were examined and approved by Le Comite de Protection des Personnes SUD-EST IV at the University of Lyon. Following the protocol approved by this committee, participants first read a description of the study procedures, about any risks or discomforts, confidentiality measures, and were provided with the contact information for the primary investigator in order to ask any questions. Compensation was described during advertising, which the participants were then reminded of. Consent was written, but anonymous, as consent would have been the only point at which personal identity would have been revealed. Participants marked either “yes” or “no” in response to the statement “I have read and understood the above consent form and desire of my own free will to participate in this study.” Participants could not continue to the rest of the study unless they had indicated “yes”. Record of consent was stored with the rest of the participant data.

### Materials pre-test

We wrote six sets of texts ostensibly authored by employees of various businesses to describe one of their new products. We used familiar concepts in order to effectively explore our cognitive hypothesis, providing plausible sets of information rather than more dramatic or outlandish claims in some urban legends and conspiracy theories, which many participants would reject out of hand in a study context. The products described were (1) a guided trek in the Amazon, (2) a data-base computer program, (3) a cooking recipe, (4) a washing machine, (5) baby diapers and (6) a seaside resort. To calibrate the different sets of stimuli used in the studies, we ran a pre-test with 55 US resident participants recruited via the Amazon m-Turk website, to rate key sentences from these texts.

For each product described, we created three versions that differed only in one key sentence, describing a potential danger (threat-related information or TRI); a negative feature of the product (NEG); or a neutral feature (NEU). For example, the participants saw the following (TRI) text displayed on the screen: “This was taken from the instructions for use manual, for installing a program on a personal computer: ‘If you press control keys during installation, the software may damage your hard disk.’”

For each such statement, we asked participants to provide 1–7 Likert-scale ratings as answers to the following questions: [a] “How useful is this statement?”, [b] “Is it negative in tone?”, [c] “Does it describe a potential danger?”, [d] “Is it written in good English?” and [e] “Does it describe some advantage of the product?”

We focused on the ratings in terms of “negative tone” and “describes potential danger”. Given the hypothesis that mention of threat-information would trigger specific intuitions of competence, we needed to check that TRI items were indeed interpreted as “describing danger”, more so than other (NEG or NEU) items. Also, we tested the extent to which our NEG items were indeed perceived as “negative in tone”, more so than NEU items. We also expected that TRI and NEG items would be judged equally “negative in tone”.

S1 and S2 Figs summarize the results, for the “includes danger” and “negative in tone” questions.

For the “includes danger” question, TRI items in all stories were rated higher than NEU items in all text-sets. Planned comparisons confirmed that the effect was significant for all text-sets, all *p*s<.001 (two-tailed). The results were mixed as regards the difference between TRI and NEG items. In four of our situations (trekking, computer, cooking, washer), the TRI items were much higher in “danger” than the NEG items. For two other stories (baby diapers and seaside resort), the difference was much smaller, as NEG items were rated relatively high in terms of “including danger”. To measure this apparent discrepancy, we evaluated the effect-sizes (Cohen’s *d*) for the difference between TRI and NEG items, in terms of “danger”, in all stories. These measures, reported on S1 Fig, confirm that the distinctiveness of our TRI items is higher in the first four stories than in the last two. There were no significant differences (all *p*s>.2) between stories in usefulness and good English (high ratings for all items).

For the “negative tone” question, the ratings were consistent with the prediction, that both NEG and TRI sentences would be rated higher than the NEU sentences (see S2 Fig). Planned comparisons confirmed that the effect was significant for all text-sets, all *p*s<.001 (two-tailed).

## Study 1

In this study, participants were asked to read and compare descriptions of three distinct products (a trekking trip, a computer program, and a cooking recipe) ostensibly provided by two different sources for each topic. The contents of the descriptions were essentially identical, save for the inclusion of threat-related content in the key version, replaced with neutral content in the control version. After reading each pair of descriptions, participants had to choose which of the authors seemed more competent.

### Methods

#### Participants

We recruited 166 US resident participants using the Amazon M-Turk website. There were 58 women. Ages ranged 18 to 70, *M* = 30.5, *SD* = 9.41. Among these 37 participants self-identified as members of an ethnic minority, and 129 as White.

#### Materials

We used the first three text-sets mentioned above, concerning (a) what to expect during a trek through the Amazon in the company of specialized guides; (b) how to install a program on a personal computer; (c) how to prepare and cook a stew of wildebeest meat (see complete texts in S1 Appendix). The key difference between the two texts, in each set, consisted in a single sentence that provided either threat-related information or neutral information, as indicated in Table 1. The rest of the texts were essentially similar, as each piece of non-key information (e.g. “this computer program will take up 100MB of your disk”) was presented in all three versions in slightly different formulations (e.g. “you will need 100MB of free space on your disk for this program”), and in a different order.

**Table 1:** Study 1, key passages that differ between the threat and neutral versions of the descriptions of three situations.

	Threat content (TRI)	Neutral content (NEU)
Trekking	[…] There are leeches that cling to your feet and can give you very serious deep burns. […]	[…] There are many species of colorful birds and flowers […]
Computer program	[…] If you press control keys during installation, the software may damage your hard disk. […]	[…] During installation the program will check that your hard disk is in good condition and report on how reliable it is. […]
Cooking	[…] If left to simmer too long the meat will turn bitter. […]	[…] You can leave all this in the fridge overnight. […]

#### Design and procedure

There were three trials, one for each of the situations: Trekking, Computer, Cooking. At each trial, participants were shown the two versions of the story (Threat and Neutral respectively), in parallel on the computer screen. After reading the two parallel versions of an explanation, participants had to answer the question, “Which of these two persons do you think is more competent, concerning the trek [/program installation/cooking recipe]?” The cover story–judging people’s presentations of a commercial product, in a competitive situation–made it natural to compare very similar versions. So the method provides an ecologically valid measure to assess the unique contribution of the key items to people’s judgments of competence. Each question was a three-alternative forced-choice item, with the two names of the different sources and “Don’t Know” as possible choices. The order of trials and side of the monitor for displaying the key and control versions, were counter-balanced across subjects. For each set of two texts, we counterbalanced which version of the text included the TRI item vs. the NEU item. After completing the three trials and answering demographic questions, participants were debriefed about the goals of the study.

### Results and discussion

Overall completion time was *M* = 180s (*SD* = 85), but a number of participants had completed the task in less than 80s, which suggested negligence. To establish a sensible cut-off point, we considered the average silent reading time for English text, that is estimated at roughly 200–400 words per minute and about 30% less for full comprehension [20,21]. Each of the text-sets used here comprised about ~250 words. So it would take the participants 122s to read all three texts-sets at the fast reading rate of 400w/m, without any time left for pondering each of the questions. This led us to exclude the results from all participants whose total completion task was under 120s, a very conservative criterion. This left 111 participants, 31 women, 23 members of minorities, ages ranging from 18 to 63, *M* = 30.5, *SD* = 9.4.

We computed two dependent variables. One was the number of participants choosing the Threat or Neutral sources as more competent, or the Don’t Know option. The second DV was a score, for each participant, of the number of choices of the Threat source over all three trials, between 0 and 3.

Table 2 summarizes the numbers of participants making the different choices. For all three text-sets, the proportion of participants choosing the TRI (threat-related) source was above chance, binomial test with a ⅓ chance of success (i.e. of choosing the TRI source), *p*<.001.

**Table 2:** Study 1, proportion of participants (%) who chose the threat-related source, neutral source, and “don’t know” for each of the situations described.

	Threat	Neutral	Don’t know
Trekking	73.0	21.6	5.4
Program	68.5	26.1	5.4
Cooking	55.9	40.5	3.6

Using the combined responses as a continuous DV out of a maximum score of 3, overall mean was *M* = 1.973, *SD* = .995, 95% CI [1.79, 2.16], which was significantly above a chance level of 1 in a one-group t-test, *t*(110) = 5.01, p<.001 (two-tailed). There were no significant effects of age, sex or ethnicity, all *p*s>.35.

Results suggest that participants intuitively associate the mention of potential threats with relatively higher competence, regarding a particular field of action, compared with non-threat-related information. In study 2 we attempted to replicate this result, using a slightly different formulation to gauge this association.

## Study 2

The results of study 1 were interpreted as supporting the hypothesis, that participants intuitively construe the communication of threat-related information as an index of competence. However, a “negativity bias” (see introduction) could make the text including threat-related information more salient than neutral text, therefore creating a response bias. To check this, we ran a replication of study 1, replacing neutral content with clearly negative content. The two versions of the stories were now of the same valence, except that one included threat-related negative information.

### Methods

#### Participants

We recruited 167 US residents from the Amazon M-Turk website, ages 18 to 76 (*M* = 32.8, *SD* = 8.5), 70 women, 125 self-identified as “White” and 42 as other ethnicities.

#### Materials

These were identical to materials in Study 1, except for the substitution of negative statements for the neutral statements used previously in control items. Instead of the NEU sentences of our pre-test (see Materials pre-test), we used NEG sentences that had rated at pre-test as significantly more “negative in tone” than the neutral ones. Table 3 lists the critical items for each text-set.

**Table 3:** Study 2, TRI and NEG items used in three text-sets.

	Threat content (TRI)	Negative content (NEG)
Trekking	[…] There are leeches that cling to your feet and can give you very serious deep burns. […]	[…]The Amazon is the poorest region of Brazil, with fewer schools, cities and roads than any of the other regions. […]
Computer program	[…] If you press control keys during installation, the software may damage your hard disk. […]	[…] The program can take a long time to master because the instruction manual is very complex. […]
Cooking	[…] If left to simmer too long the wildebeest meat will turn very bitter. […]	[…] Some people don’t like this kind of stew because it looks gray, which they don’t find appetizing. […]

#### Design and procedure

Identical to Study 1.

### Results and discussion

Using the same criteria as in Study 1, we excluded the data from participants whose completion time was under 120s. This left 129 participants, ages 18 to 76 (*M* = 30.2, *SD* = 11.4), 37 women, 19 minorities.

The proportions of participants’ choices are summarized in Table 4. For all three stories, the proportion of participants choosing the threat-related source was significantly above chance, binomial test with a ⅓ chance of success (i.e. of choosing the threat-related source), all *p*s<.001.

**Table 4:** Study 3, proportion of participants (%) who chose the threat-related source, negative source, and Don’t Know for each of the vignettes: trekking, computer program and cooking recipe.

	Threat	Negative	Don’t Know
Trekking	71.3	24.8	3.9
Computer program	72.9	24.0	3.1
Cooking	60.5	34.9	4.7

Using the combined responses as a continuous DV out of 3, the overall score was *M* = 2.047, *SD* = .995, 95% CI [2.042, 2.051], different from the chance level 0f 1, *t*(128) = 7.59, *p*<.001 (two-tailed). There were no significant effects of age, sex or ethnicity, all *p*s>.40.

This suggests that the preference for threat-related information in study 1 was not caused by the difference in valence between threat-related and neutral content.

## Study 3

To check that the association between threat-information and competence in studies 1–2 really stemmed from the potential danger contained in our critical items, we replicated study 1, using a new set of texts, this time concerning (a) a seaside resort, (b) a new kind of baby diapers and (c) a new washing machine. These texts had, as in the previous studies, been pre-tested for potential danger and negativity. Only one of these new sets of texts, about the washing machine, showed a significant difference in ratings between Negative and Threat items at pre-test (as described above). We predicted that participants would choose the threat-source in that particular situation, due to the perceivable difference in danger, but not in the other ones, due to the absence of perceivable danger.

### Methods

#### Participants

We recruited 175 US residents using the Amazon M-Turk website, ages 18 to 68 (*M* = 34.3 *SD* = 12.5), 101 women and 74 men, 127 self-identified as “White” and 48 as minority ethnicities.

#### Materials

We used three text-sets mentioned in Pre-test section, concerning (a) a seaside resort, (b) a new kind of baby diapers and (c) a new washing machine. Table 5 summarizes the critical differences between these text-sets.

**Table 5:** Study 3, key sentences that differ between the threat and negative versions of the descriptions of three situations.

	Threat content	Negative content
Seaside resort	[…] The gates are locked after 12am so you may be locked out unless you warn the staff of the time you will come back. […]	[…] The resort is located on a narrow dirt road, and is less easily accessible than other places on the coast. […]
Diapers	[…] If you fold the sides first the baby’s pee may leak outside the diaper, and cause rashes and infections. […]	[…] The adhesive straps at the end of the side folds often do not stick, so you may have to start all over again. […]
Washer	[…] The very high speed Ultra-Spin may damage delicate fabrics, use only regular spin speed for these clothes. […]	[…] The Mehlen 250, because of all these features, is of course more expensive than regular washers with the same washing volume. […]

#### Design and procedure

Identical to Study 1.

### Results

As in previous studies, we excluded data from participants whose completion time was under 120 s. This left 128 participants, ages 18 to 68, (*M* = 37, *SD* = 12.6), 75 women, 35 minorities.

The participants’ choices of sources are reported in Table 6 below. The pattern of choices was different for the seaside and diaper stories, on the one hand, and the washer story on the other. For the latter story, more participants chose the threat-source as more competent, a significant difference binomial test with ⅓ success rate, *p*<.001. The association was not significant for the other two stories, both *p*s>.8 for the binomial test. The difference between the stories was statistically significant, *χ* ^2^(2) = 16.1, *p*<.001.

**Table 6:** Study 3, proportion of participants (%) who chose the threat-related source, neutral source, and Don’t Know for each of the different situations described.

	Threat	Negative	Don’t Know
Seaside resort	42.5	57.5	0.0
Baby diapers	48.8	51.2	0.0
Washer	66.9	33.1	0.0

These results would suggest that the association between threat-information and competence in studies 1–2 was indeed driven by the mention of potential danger in the threat-source versions of the different texts. For two text-sets, both versions (NEG and TRI) were identified at pre-test as relatively close in “potential danger” ratings. This resulted in chance performance when choosing for the more competent source. By contrast, there was a clear choice of the threat-source when considering the one text-set for which there was a greater difference in “danger” rating between the two versions at pre-test.

## Study 4 [TRIcomp05 –TRI better or NEU worse?]

In studies 1–3, the source providing threat-related information was consistently judged more competent than the source that did not provide such information. This could support the notion that threat-information is intuitively seen as an index of competence, reliability, etc. However, a slightly different interpretation is possible, in which the effect is driven by a negative estimation of the non-threat source. Specifically, since the participant is informed (by one of the sources) of a potential threat (e.g. the leeches in the forest or the computer freeze), he or she may form the impression that the *other* source, which did not mention that threat, was negligent or irresponsible.

To test this hypothesis, we used two of our pre-tested text-sets in a modified protocol, in which participants read the threat and non-threat versions of the story sequentially, and provided a Likert scale estimate of how helpful the information was after reading each version. For one text-set, threat came before non-threat information and *vice-versa* for the other. Our reasoning was that, if the threat-source was seen as intrinsically more valuable than the neutral source, the rating of threat-source would be much higher when threat-information came in second position (after the participant had read a neutral version) than if it came in first position (in which case participants could not detect the threat-information as a distinctive feature). By contrast, if negligence in the neutral source drove the effects observed so far, we should observe a large drop in ratings between neutral source in first position (probably judged useful, with no comparative basis for judgment) and neutral source in the second position (judged negligent, as it fails to mention a danger that the participant now knows about). Obviously, the design allows for both processes to occur simultaneously.

### Methods

#### Participants

We recruited 105 US residents using the Amazon M-Turk website, ages 19 to 73 (*M* = 33.6, *SD* = 12), 38 women and 67 men, 86 self-identified as “White” and 19 as minority ethnicities.

#### Materials

We used the “trekking” and “computer program” texts of study 2, followed by the question: “How useful do you think this description was?” instead of the forced-choice of study 1.

#### Design and procedure

Participants were instructed in the same way as in previous studies. For each situation, they first read the text introducing the situation, then one of the descriptions (either threat-information or non-threat), followed by a prompt for their estimate of usefulness, using a 1–7 Likert scale. They then read the alternative description (non-threat or threat) of the same situation, followed by the same prompt. This was repeated twice. The threat/non-threat order, and the order of stories, were counter-balanced across participants.

### Results and discussion

We excluded data from participants with completion rate under 120s. This left 69 participants, ages 19 to 73 (*M* = 36.4, *SD* = 13.6), 26 women and 15 members of ethnic minorities.

Average ratings for the two sources are illustrated in S3 Fig.

A repeated measure ANOVA showed a significant main effect of information type (threat vs. neutral), *F*1,68 = 22.2, *p*<.001, partial η^2^ = .25; no significant main effect of serial position (first vs. second text), *F*1,68<1; a significant information type * serial position interaction, *F*1,68 = 14.3, *p*<.001, partial η^2^ = .17. There was no significant effect of sex, *F*1,68 = 1.1, *p* = .35; a trend to an effect of ethnicity, *F*1,68 = 3.1, *p* = .09; no effect of age, *p* = .30, and no further interactions.

Planned comparisons showed a significant difference between ratings of threat-first and threat-second texts, *t*(68) = 3.25, *p* = .002, 95% CI [.21, .88]; for neutral first compared to neutral second texts, there was a trend to a significant difference above the conventional α, *t*(68) = 1.89, *p* = .062, 95% CI [.02, .68]. Confirming this, the effect-sizes for these two tests are different, Cohen’s *dz* = .49 for the threat-items (between 1^st^ and 2^nd^ presentations), and *dz* = .28 for neutral items (again between 1^st^ and 2^nd^ presentations).

These results suggest, first, that the competence impression observed in studies 1–3 is a contrastive effect that obtains when participants are confronted with otherwise highly similar threat-including and non-threat-including sources on the same topic. In this study, due to the sequential presentation of the sources, we could measure whether threat- would be rated higher than non-threat information, independently of this contrast. But that was not the case, as the initial ratings for threat and non-threat sources (*M* = 5.4 and *M* = 5.5 respectively) were not different.

Results also suggest that this contrastive effect may be driven both by a devaluation of the neutral source and by a higher evaluation of the threat source. If the neutral source was devalued as negligent after hearing of potential threats, the ratings for the neutral source in second position should be significantly lower than they are for neutral source in first position–as the information from that second neutral source would be judged as clearly insufficient. We observed a trend in that direction. By contrast, if the threat source is seen as more informative than the neutral source, we should expect it to be rated significantly higher when it can be contrasted to a neutral source, which is only possible when it is read as the second text. We should therefore expect a significantly higher rating of the threat source as second text, compared to the threat source as first text, which is what we observed.

## Study 5 [TRIcomp07 –not globally positive]

Results of studies 1–4 suggest that sources conveying threat-related information are judged competent in contrast to other sources. We hypothesized that this may contribute to the cultural spread of threat-related information. But this effect may be due, not just to a specific effect on competence, but to an overall positive impression provided by the communication of threat-information. To judge whether the contrastive effects observed so far are driven by a specific intuition of competence, we replicated study 1 with a set of different questions, asking participants, not just about competence, but also about perceived honesty and pleasantness of the sources. If threat-information suggests competence in a narrow way, then it should have no positive effect on pleasantness–in fact the opposite should be expected, as the information in question is unpleasant and may contaminate the source. There should be no effect on honesty, as neither threat- nor neutral-information provides cues for deception or dishonesty.

### Methods

#### Participants

We recruited 106 US residents using the Amazon M-Turk website, ages 19 to 65 (*M* = 35.5 *SD* = 12.2), 56 women and 50 men, 73 self-identified as “White” and 35 as minority ethnicities.

#### Materials

Texts identical to Study 1.

#### Design and procedure

Identical to Study 1, except that each pair of side-by-side texts was followed by three questions: [a] which of these do you think is more competent? [b] which of these do you think is more honest? And [c] which of these do you think is more pleasant as a person? Each question was a three-alternative forced-choice, with the two sources and “don’t know” as possible choices.

### Results

We excluded participants whose completion time was under the minimum reading time. This left 68 participants, ages 19 to 65, *M* = 38.6, *SD* = 13.3, comprising 39 women and 19 members of ethnic minorities.

As in previous studies, we computed both the numbers of participants making each choice in each situation, and a combined score with the number of times they selected the threat-source over three trials.

Choices are summarized in S4 Fig, showing that participants tended to choose the threat-source as more “competent” than the others, that the scores for “honest” were closer to chance for cooking and program but favored the threat source for trekking; participants did not choose the threat-source as the “pleasant” one. Results significantly different from chance, on a binomial test with ⅓ as p(success), are marked ** on the chart.

The combined score was computed by adding all the trials in which the participant had chosen the threat-source over the other sources. Different one-group t-tests compared the means for the different criteria (competent, honest, pleasant) with a chance level of 1.5, as in previous studies. The results show significant deviations from chance for both “competent” and “pleasant”, but a chance result for “honest”. One-group t-test results with p<.001 (two-tailed) are marked *** on S5 Fig. There was no effect of sex or ethnicity, both *p*s>.10, or correlation between age and responses, *p*>.20.

These results replicate the finding of studies 1–4, as the communication of threat information is associated with competence, when compared with sources that do not communicate such information. They also reflect that this effect is driven by a specific intuition of competence, not by a general “positive glow” associated with the particular source. Participants were at chance or favorable to the threat-source when judging honesty–the choice of the threat-source would make sense in a commercial context, where customers appreciate to be warned of potential problems with the product. Participants generally judged the threat-conveying source as less pleasant than the others, probably as an effect of the negative information contaminating the source.

## General Discussion

Humans rely on other humans for information, coordinated activities, to help avoid dangers, and to confront threats when they appear. This reliance also comes with risks, such as acting on the basis of incorrect information, and selecting incompetent partners. A large body of research examines how characteristics such as authority, physical attractiveness, and markers of in-group coalition influence the believability of what that person communicates [22]. The current research expands on these ideas to examine the possible role of the content of the information in an interaction, and how this information leads the target to make judgments about the competence of the information source.

There is good evidence to support the notion that due to error management, people are likely to believe threat related information [14–16]. Congruent with these results, the present studies support the idea that sources of threat related information are viewed as more competent and useful. This competence effect may feed back into the transmission of cultural information. By taking sources of information about hazards more seriously than others, listeners would also contribute to the cultural spread of threat- and precaution-related information. Though this series of studies relied on a North American population, we predict that the same pattern would be found in diverse cultural environments, given the centrality of threat to cognitive processing and the importance of good sources of information in the evolutionary context, though this remains to be tested.

Studies 1–5 demonstrated a clear pattern whereby a source of threat-related information was judged more competent than a source of equivalent information with no mention of potential danger. Successive replications showed that the effect (a) occurred with materials describing very different products and situations, (b) that it was not driven by the negative valence of threat information (studies 2, 3), (c) that it is at least partially driven by a positive evaluation of the threat-source (study 4), and (d) that it is specifically about competence rather than being grounded in an overall positive “glow” around the threat source (study 5). Study 4 also confirmed (e) that the competence impression occurs in the context of a contrast between the two sources. These results support the notion that the transmission of threat-related information can improve the perception of the source as competent and therefore a potentially useful partner. Future studies should investigate the longevity and the generality of the effect.

Obviously, the dangerous features described in our stories may seem trivial compared to the usual subject-matter of many cultural rumors (murders, penis-snatching, poisoning wells and kidnapping children). We conjecture that the competence effect observed here may prove just as strong or stronger when the threat described is more serious, although this of course requires that the rumor is held to be true. Only naturalistic studies could validate that conjecture.

In a speculative manner, the present results may allow us to put forward an interpretation of the cultural success of threat-related material that is more specific than a simple “negative bias”. First, it seems that the ecology of human evolution comprised many potential dangers, against which organisms had fewer defenses than their predecessors [12]. For instance, evolving a generalist diet resulted in an increased vulnerability to pathogens; depending on cooperation for survival made humans vulnerable to status loss. Second, human defenses against such threats consist for a large part in socially transmitted information, in other agents pointing to hazards and providing precautionary information [23]. Third, human communication allows for useful information transfer but also for deception as well as straightforward nonsense [24]. Having in one’s social environment some sources of information about potential danger would be precious, and one would be motivated to weigh such information above the rest, and to value the sources accordingly–a valuation that would be reflected in specific judgments of “competence” expressed by the participants in our studies. This competence effect may feed back into the transmission of cultural information. Furthermore, we may speculatively suggest that information about potential danger does not invite testing, so sources of incorrect threat related information may retain their reputational advantage. By taking sources of information about hazards more seriously than others, listeners would also contribute to the cultural spread of threat- and precaution-related information.

## Supporting Information

S1 Appendix Complete texts used in studies 1–3. (DOCX)

S1 Fig Pre-test. For each story, ratings of TRI (threat), NEU (neutral) and NEG (negative) sentences, in terms of “mentions danger”. Brackets include effect-size (Cohen’s d) for the comparison between TRI and NEG items.
(TIFF)

S2 Fig Pre-test. For each text-set, ratings of TRI (threat), NEU (neutral) and NEG (negative) sentences, in terms of “negative tone”.
(TIFF)

S3 Fig Study 4. Average “usefulness” rating of the neutral and threat sources, as either 1^st^ or 2^nd^ text presented (error-bars: 95% CI).
(TIFF)

S4 Fig Study 5 Proportion. Proportion of participants (%) who chose the threat or neutral source or “don’t know” as more competent, honest and pleasant in three different text-sets (**: p<.001).
(TIFF)

S5 Fig Study 5 Combined Scores. Combined scores for choice of the threat-source (out of maximum 3) as the source more likely to be competent, honest and pleasant (Error-bars: 95% CIs, ***: p<.001).
(TIFF)
