# Supplementary material for: Threat-Related Information Suggests Competence: A Possible Factor in the Spread of Rumors
Source: PLoS One. 2015 Jun 10;10(6):e0128421. doi: 10.1371/journal.pone.0128421 (PMC4464524; doi:10.1371/journal.pone.0128421)
Supplement: S1 Appendix — (DOCX) [file pone.0128421.s001.docx]

Threat-related information suggests competence:
A possible factor in the spread of rumors

Pascal Boyer & Nora Parren

# Supporting Information

Appendix – Complete texts used in studies 1-3

| Trekking, threat version | Trekking, non-threat version |
| --- | --- |
| These two persons have been trained to serve as guides on a trek through the Amazon, specially for the BrazilTrek® 10 day trek through the Amazon rain forest. They both explain to you, in the text below, what to expect in this kind of trip. After reading their explanations, you will have to judge which one is better. Read attentively: | |
| MARIA:  “Be prepared for the heat (always above 100F) and humidity (close to 100%). Wear light clothes. We will walk for about 6 miles each day. In the forest this can take about 5 hours.  Be aware that there are leeches that cling to your feet and can give you very serious deep burns. But if you wear high rubber boots you will be fine.  We will sleep on mats that most travelers say are pretty comfortable. Each evening we eat at the village refuges. Get in touch before the trek if you have any other questions.” | EMILIA:  “In general, we will walk for about 5 hours a day, the refuges are about 6 miles from each other. At the refuge you will find your mat for a comfortable night, and of course food and water.  Very important: There are many species of colorful birds and flowers so you will have lots of opportunities for great pictures!  It will be pretty hot and humid, 100F or more, and you will feel you are in a greenhouse. Only wear light clothes, given how hot it is. I will be happy to give you more information if you need any.” |

| Computer program, threat version | Computer program, non-threat version |
| --- | --- |
| These two computer programmers, Scott and Adam, are data-base specialists. They work for the company that produces the DBXcess® data-base software. They both explain to you, in the text below, how to install the program. After reading their explanations, you will have to judge which one is better. Read attentively: | |
| SCOTT:  “The DBXcess® data-base will require about 100 Mbytes of disk space, and 200 Mbytes during installation. You will need to download the installer from our website, OR insert the DVD in your computer. Then you will have to choose whether you want a standard install or a custom one with different settings.  The crucial thing is that if you press control keys during installation, the software may damage your hard disk. But if you install it from a DVD there is no such risk.  The process should not take more than 15 minutes with the DVD, 25 minutes from the website.” | ADAM:  “There are two ways to install the DBXcess® data-base program, either from the DVD (about 15 minutes) that you received with your license, or from our website (about 25 minutes). Your hard disk should have about 100 Mbytes of free space for the program, and twice as much free space during installation.  People should know that during installation the program will check that your hard disk is in good condition and report on how reliable it is.  Most people use the standard version of DBXcess® but you can customize the program during installation.” |

| Cooking, threat version | Cooking, non-threat version |
| --- | --- |
| Mario and Owen just had a cooking lesson at the Great Desert restaurant in Johannesburg, South Africa, where they learned how to make the traditional South-African wildebeest and yam stew. They both explain to you, in the text below, how to make that stew. After reading their explanations, you will have to judge which one is better. Read attentively: | |
| MARIO:  “For 6 people, you will need about 2lbs of wildebeest meat (not too lean). Cut it in roughly 1” pieces. Brown them on high heat for about five minutes. Then pour them into the casserole with 2lbs of sliced yams, and seasoning (salt, pepper, coriander), with two sliced onions. Cover with water mixed with a glass of white wine. Keep simmering for about two hours.  Remember this: If left to simmer too long the meat will turn bitter. But if you add onion to the stew it will be just fine.  You can find wildebeest meat in all markets in South Africa, and a few places in the United States.” | OWEN:  “This stew is a simple mixture of sliced yams, salt and pepper and coriander, and nice pieces of wildebeest meat, not too lean. You find that in some stores in the US, and everywhere in South Africa. Take about 2lbs of meat, and the same amount of yams. You should add one or two onions. You should brown the meat first on high heat, before mixing it with the other ingredients and a glass of white wine.  What you should remember is that you can leave all this in the fridge overnight. They say it is even better that way.  Keep simmering for two hours or so.  Serves 6.” |

| Seaside Resort, threat version | Seaside Resort, non-threat version |
| --- | --- |
| These two employees of Rising Sun Resort™ describe the place for potential visitors. | |
| RACHEL:  However long you stay at Rising Sun Resort, you will wish it was longer! We have direct access to a two-mile long sandy beach.  The bungalows all have a direct view of the sea. The three resort restaurants with distinct themes will cater to all tastes, from individuals to family meals to romantic dinners. There is a bar and a discotheque.  Note that the gates are locked after 12am so you may be locked out unless you warn the staff of the time you will come back.  There is a children’s club where young visitors can discover or practice wind-surfing, sailing and beach volley-ball with trained counsellors. | SANDY:  The Rising Sun Resort will be happy to accommodate all your needs! Children and youngsters may wish to join the beach clubs, where our trained counsellors will organize sports activities, including beach-volleyball, sailing and wind-surfing.  Remember that the resort is located on a narrow dirt road, and is less easily accessible than other places on the coast.  Your bungalow will be located on the beach and look out on the ocean. You can walk straight to our long sandy beach. For dinner, you have a choice of three themed restaurants with a distinct ambience. Family, couples and individuals will all find a welcoming place to eat. For drinks or dancing, we have a bar and a discotheque. |

| Diapers, threat version | Diapers, non-threat version |
| --- | --- |
| These two nurses have been asked to write advice to new parents on how to choose and change diapers. Please read the two texts and tell us how competent you think these persons are. | |
| BRENDA:  There are many brands of diapers. Do not think that the more expensive brands are better. They are generally the same as store brands.  Contact between skin and diaper can be painful. Rub the baby’s bottom with soothing ointment every time you see a rash. Use a wet cloth or towelette to clean the infant’s bottom and genital area.  Place the diaper wide open on the bed or table, and place the baby in the right position, facing you. Fold the bottom part first, then the two side flaps on top of that.  Always choose the right size, they are numbered from 1 to 6 and follow the child’s growth. Change the diaper each time the child has soiled the diaper.  Note that if you fold the sides first the baby’s pee may leek outside the diaper, and cause rashes and infections.  Disposable and re-usable diapers are the same for the baby. Some parents prefer re-usable ones because they are better for the environment, but require more work. | LYNN:  Many parents are confused whether they should choose disposable diapers or the old-style re-usable ones, which are of course more environment-friendly. From the baby’s viewpoint, there is no difference at all.  As far as brands are concerned, diapers from your store brand (e.g. Walmart, Target, Walgreens) and from the more famous brands, are practically identical.  Babies should be changed very often, in fact every time you notice they need change. After you open the diaper, be sure to clean the entire are with a wet cloth or towelette. Place the infant on his back, on the open diaper, close the bottom flap first by pulling it up, then fold both side flaps and use the adhesive straps to secure the diaper in place.  Note that the adhesive straps at the end of the side folds often do not stick, so you may have to start all over again.  Diapers often cause diaper rash, which is painful. It will be very visible after cleaning up the infant’s bottom. You should rub the affected area with a soothing cream. |

| Washer, threat version | Washer, non-threat version |
| --- | --- |
| These two engineers were involved in designing the new Mehlen™ washing machine. Here they explain its main features. Read both descriptions and tell us how competent you think these persons are. | |
| CHUCK  The new Mehlen 205 is the most advanced washer we have produced yet.  There are more than 2000 combinations of time, temperature, spinning speed and detergent intake.  One advanced feature is the ultra-high speed spin cycle that makes spinning much shorter, but does not consume more electricity than regular spin cycles.  Note that the very high speed Ultra-Spin may damage delicate fabrics, so be sure to use only regular spin speed for these clothes.  The main innovation concerns the optimal use of detergent and water. We have designed a new washer body, in which the rotation is inversed and the washing load is moved up and down by a plastic blade. This ensures that every inch of fabric is in contact with the detergent and then with the rinsing water.  There are special cycles for extremely delicate fabrics, with spinning as low as 30 rpm and a very gradual heating of the water. | LENNY  This is the Mehlen 250, we have designed as a revolutionary washer with many new features.  The Mehlen 250, because of all these features, is of course more expensive than regular washers with the same washing volume.  To achieve a thorough detergent penetration and rinsing, we have designed a revolutionary system in which the washing load is both rotated in changing directions every few seconds, and pushed up and down by a plastic blade.  There are cycles for instance for ultra-delicate fabrics, which not only heat the water very slowly but also slow down the spinning speed.  At the opposite end, we also provide the fastest spinning cycle ever in domestic washers, which goes at about twice the maximum speed of regular machines, without using more power.  The Mehlen 250 provides more than 2000 combinations, to adapt to all needs in terms of heat, load and detergents |
