# Supplementary figures and images for: Threat-Related Information Suggests Competence: A Possible Factor in the Spread of Rumors
Source: PLoS One. 2015 Jun 10;10(6):e0128421. doi: 10.1371/journal.pone.0128421 (PMC4464524; doi:10.1371/journal.pone.0128421)

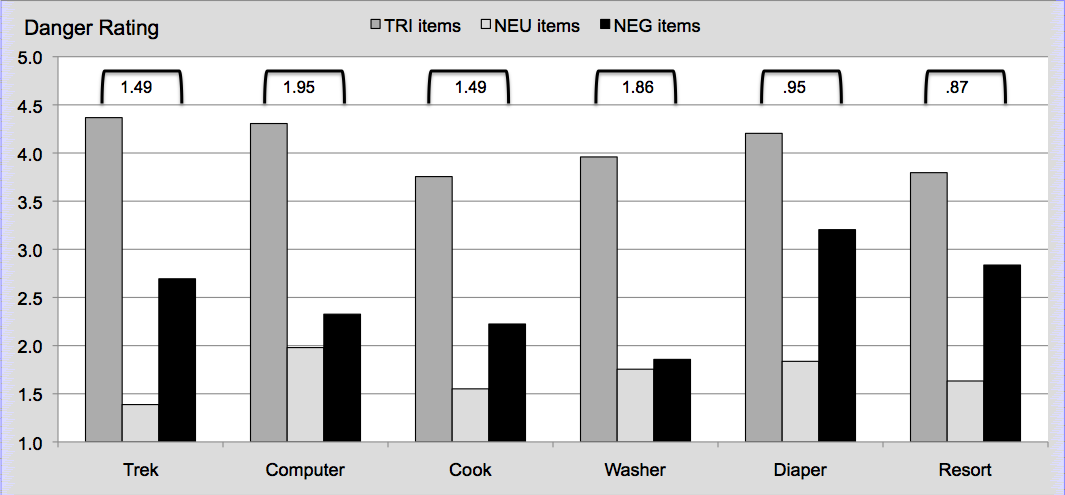

Supplement: S1 Fig — For each story, ratings of TRI (threat), NEU (neutral) and NEG (negative) sentences, in terms of “mentions danger”. Brackets include effect-size (Cohen’s d) for the comparison between TRI and NEG items. (TIFF) [file pone.0128421.s002.tiff]

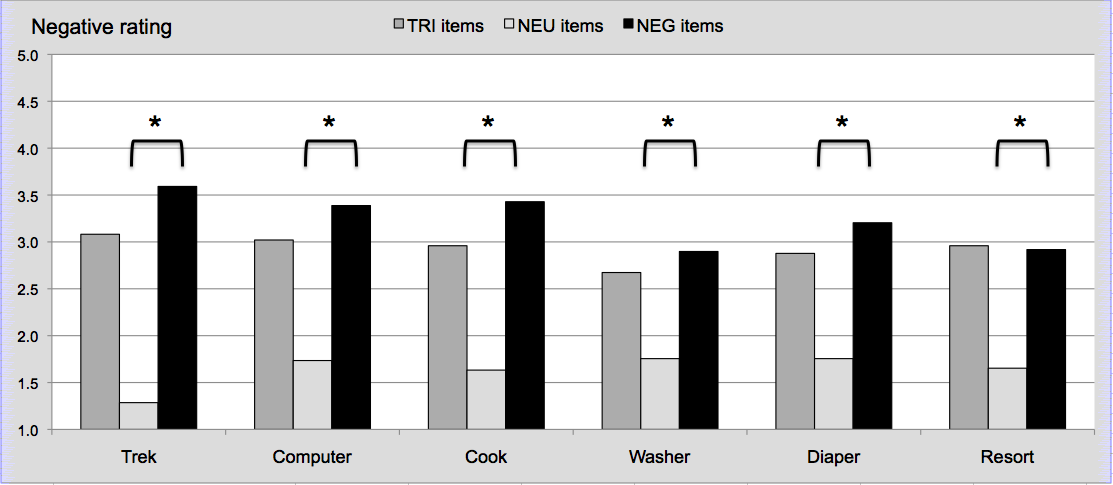

Supplement: S2 Fig — For each text-set, ratings of TRI (threat), NEU (neutral) and NEG (negative) sentences, in terms of “negative tone”. (TIFF) [file pone.0128421.s003.tiff]

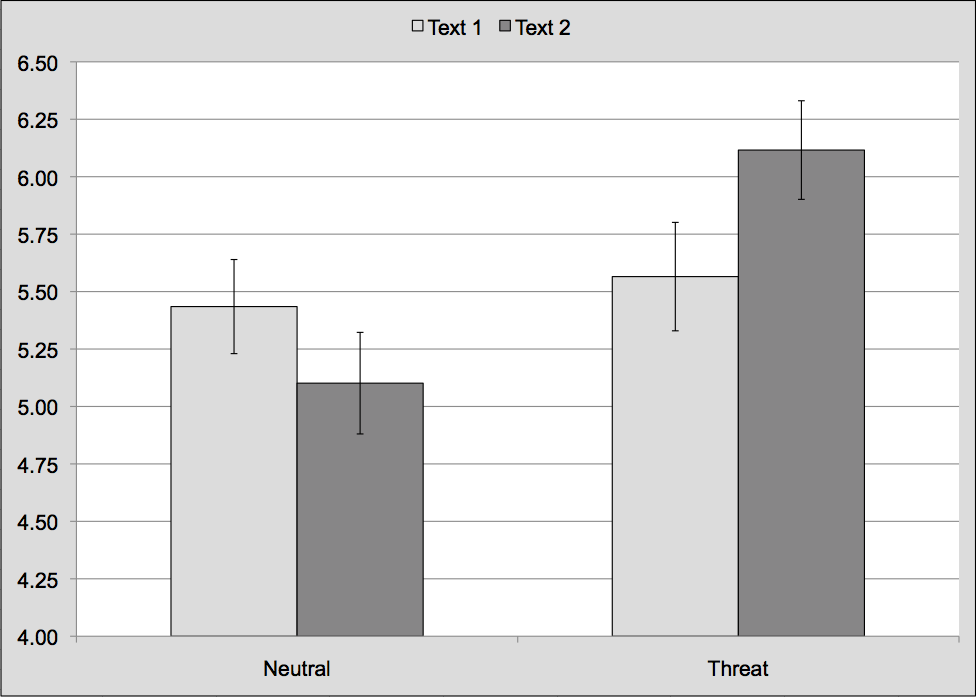

Supplement: S3 Fig — Average “usefulness” rating of the neutral and threat sources, as either 1st or 2nd text presented (error-bars: 95% CI). (TIFF) [file pone.0128421.s004.tiff]

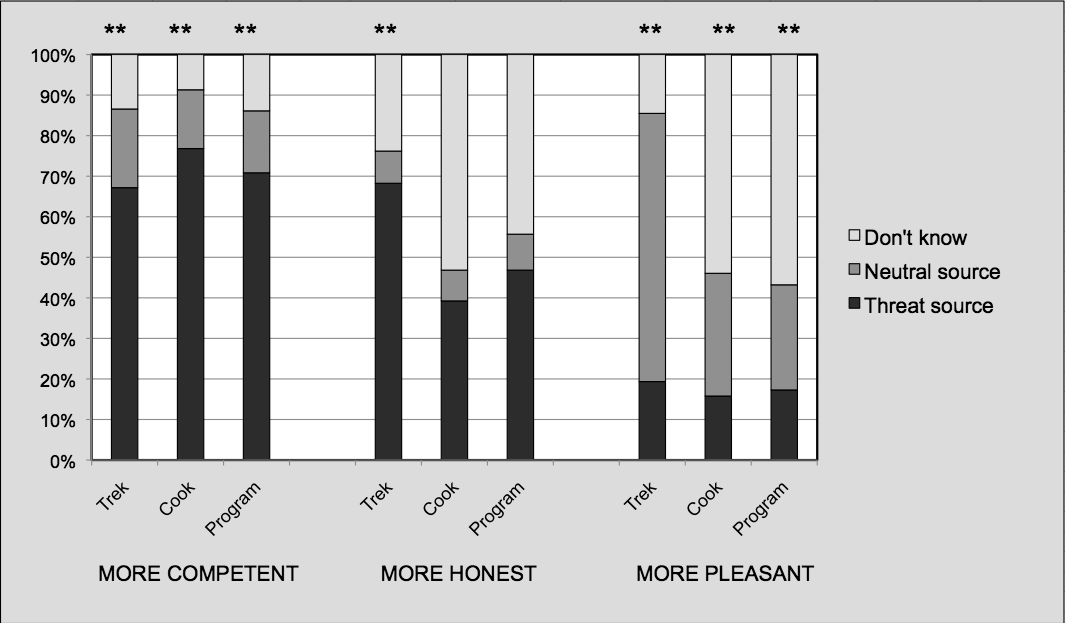

Supplement: S4 Fig — Proportion of participants (%) who chose the threat or neutral source or “don’t know” as more competent, honest and pleasant in three different text-sets (**: p<.001). (TIFF) [file pone.0128421.s005.tiff]

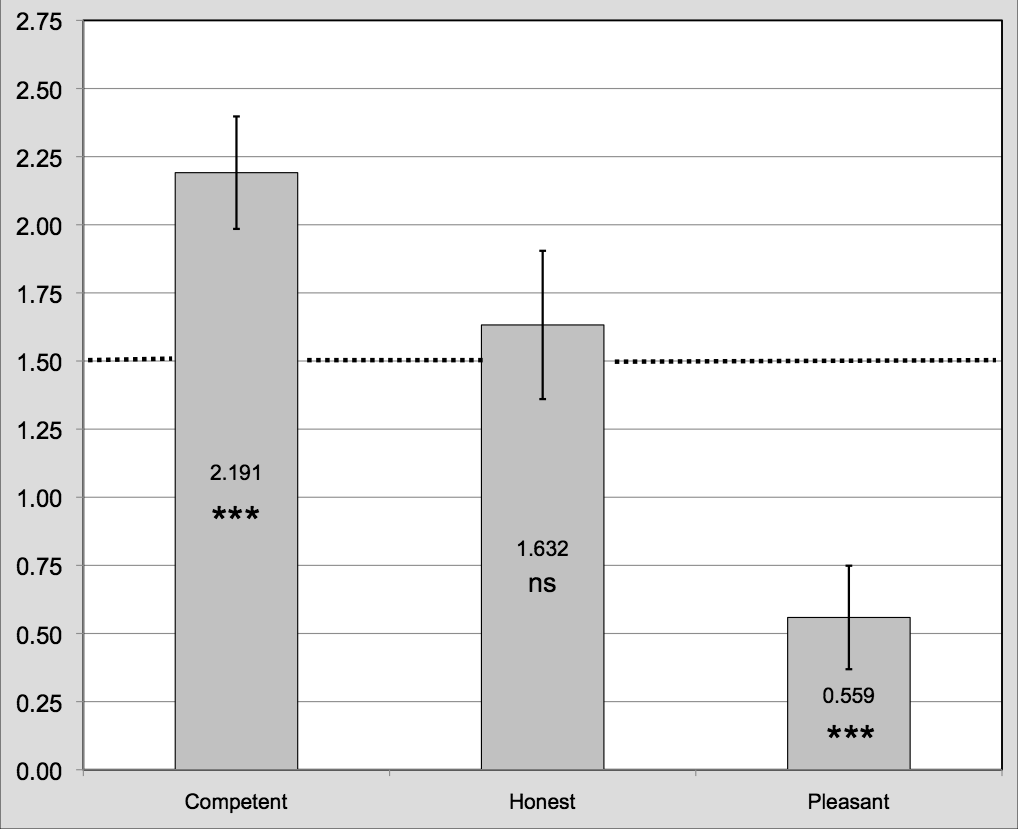

Supplement: S5 Fig — Combined scores for choice of the threat-source (out of maximum 3) as the source more likely to be competent, honest and pleasant (Error-bars: 95% CIs, ***: p<.001). (TIFF) [file pone.0128421.s006.tiff]
